# Molecular dynamics study of the effect of extended ingrain defects on grain growth kinetics in nanocrystalline copper

**DOI:** 10.1038/s41598-020-79861-3

**Published:** 2021-01-13

**Authors:** Vladimir V. Dremov, Pavel V. Chirkov, Alexey V. Karavaev

**Affiliations:** Federal State Unitary Enterprise “Russian Federal Nuclear Center—Zababakhin All-Russia Research Institute of Technical Physics”, 13 Vasiliev st., Snezhinsk Chelyabinsk Region, 456770 Russia

**Keywords:** Nanoscale materials, Structural properties, Materials science, Theory and computation, Atomistic models

## Abstract

The paper presents results of a large-scale classical molecular dynamics study into the effect of ingrain defects on the grain growth rate of face centered cubic nanocrystalline material under thermal annealing. To do this, two types of virtual MD samples are used. The samples of the first type are constructed artificially by filling Voronoi cells with atoms arranged in fcc lattice essentially with no ingrain defects. The other samples are obtained by natural crystallization from melted material and contain numerous extended ingrain defects. These samples with a high concentration of ingrain defects imitate nanocrystalline material produced by severe plastic deformation via high pressure torsion or equal channel angular extrusion. The samples of both types are subjected to long-time zero pressure isothermal annealing at $T\approx 0.9T_m$ ($T_m$ is melting temperature) which leads to grain coarsening due to recrystallization. Direct molecular dynamics simulations of the annealing of different samples show that at the same conditions recrystallization goes two times faster in the samples with a high concentration of extended ingrain defects than in the defect-free samples. That is, to increase the thermal stability of nanostructured material the technologies used for forming nanocrystalline structures should be developed so as to avoid the thermomechanical treatment regimes leading to the formation of structures with high concentration of ingrain defects.

## Introduction

The properties of ultrafine-grained materials differ greatly from those of conventional ones. In particular, these materials reveal enhanced strength properties (see review paper^1^) as well as enhanced resistance to radiation damage^2^. That is why such materials are considered as promising for many technical applications including nuclear technologies. On the other hand, extreme conditions may cause degradation of their unique properties. In particular, high temperatures cause rapid grain coarsening, and a search for efficient technologies that could provide for higher thermal stability is an urgent need in materials science.

From the numerical simulation point of view, nanocrystalline materials provide the reliable bridge between experiment and direct atomistic modeling. The grain size of $\sim 1\upmu$m is a quite available scale for state-of-the-art massively parallel classical Molecular Dynamics (MD) simulations. Classical MD simulations are only based on the semi-empirical model of interatomic interactions and give proper results if the empirical interatomic potential is properly parameterized. Starting from the pioneering works^3–10^, various properties and phenomena in nanocrystalline solids were thoroughly investigated within MD studies (see recent papers^11–14^). Most of those MD studies dealt with a local structure and the properties of grain boundaries (local stresses, defect mobility under external stress, emission of dislocations by grain boundaries and their interaction with dislocations *etc.*) and with the elastic-plastic behaviour of nanocrystalline materials under external mechanical stresses including intense shock-wave loading. So, the technique of simulations developed in^15–17^ allowed direct modeling of the elastic-plastic nanocrystalline material response to quasi-static loading and calculation of the yield stress.

Atomistic simulations also provide an opportunity to study directly various factors affecting the thermal stability of nanocrystalline solids. For example, Okita et al.^18,19^ investigated grain growth kinetics in bcc iron crystallized from the melt with the main focus on the deviation of the growth rate from ideal one due to anisotropic grain boundary properties. Another study by Holm and Foiles^20,21^ was performed to model the grain growth in fcc Ni with the purpose of revealing reasons for its stopping even in high purity materials. The authors^18–21^ paid great attention to the grain boundaries while the samples were essentially free of ingrain extended defects.

In the present work we model the grain growth during thermal annealing with the focus on the effect of extended ingrain defects on the recrystallization rate (grain coarsening rate) of nanocrystalline fcc copper. To do this two kinds of samples were constructed: the samples essentially free of defects except grain boundaries and the samples containing a lot of extended ingrain defects—numerous nanotwins and relatively rare stacking fault planes. The samples of the second kind are aimed to imitate nanocrystalline material produced by severe plastic deformation, for example, by high pressure torsion or equal channel angular extrusion.

MD simulations presented here were carried out using two massively-parallel classical MD codes, MOLOCH^22^ and LAMMPS^23,24^. For sample structure analysis we used orientation analyses by Polyhedral Template Matching (PTM)^25^ implemented in scientific data visualization and analysis software for atomistic simulations, OVITO^26–28^ and Adaptive Template Analysis (ATA)^29^ which allows precise, high confidence recognition of crystal structures and various defects in samples at elevated temperatures up to the melting point. To describe interatomic interactions the Embedded Atom Model (EAM) potential for copper^30^ is used. All simulations of crystallization, thermalization, and recrystallization were carried out for *NPT*-ensembles at zero pressure and various temperatures using Nose-Hoover^31,32^ thermostat in periodic boundary conditions in the cartesian directions.

## Virtual nanocrystalline samples generation

Atomistic simulations of recrystallization process were carried out with submicrocrystalline samples of copper. For atomistic simulations, it is crucial to construct virtual samples relevant to the problem under consideration. The detailed description of the technique used for the construction of virtual nanocrystalline samples is given in paper^16^. Briefly the procedure is as follows. There are two options to construct virtual nanocrystalline samples. The first one is to subdivide the simulation box into a number of Voronoi polyhedra cells and fill them with atoms arranged in the desired crystal lattice. The mutual crystal orientation in the polyhedra may be chosen as random or having a particular order. A special procedure is applied to provide for proper sintering of grains at boundaries^16^ to allow the use of periodic boundary conditions in all directions.

**Figure 1:**
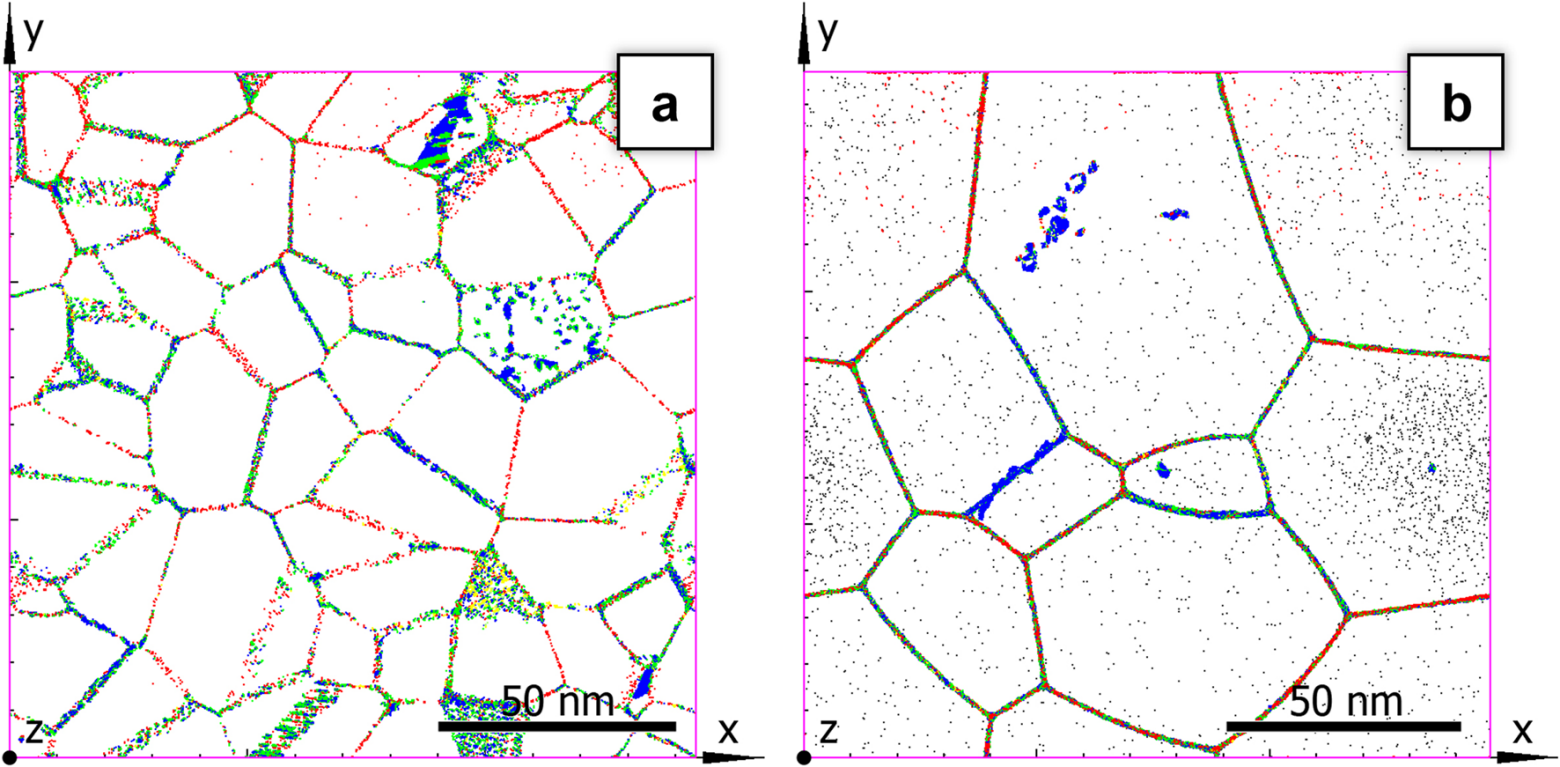
Nanocrystalline sample generated by Voronoi polyhedra filling (V-type sample): (**a**) just after the construction, (**b**) after 50 *ns* of annealing at $T=0.904T_m$. Grain boundaries are of mixed colours, the blue colour is for atoms in stacking faults and stacking faults tetrahedra. Atoms belonging to the fcc structure are not shown.

The advantage of this option is the construction of samples composed of nanocrystallines which have the required average size and are almost free of ingrain defects (see Fig. 1). One can see that the grains are “transparent” because atoms belonging to fcc structure are not shown, but a number of stacking faults and vacancy clusters (which transform in copper into stacking faults tetrahedra^33^) are formed during the procedure but their overall concentration is low. Using Voronoi filling one may choose the number of crystalline grains, their average size and mutual orientation. The virtual samples prepared with this technique are designated hereafter as V-type samples.

**Figure 2:**
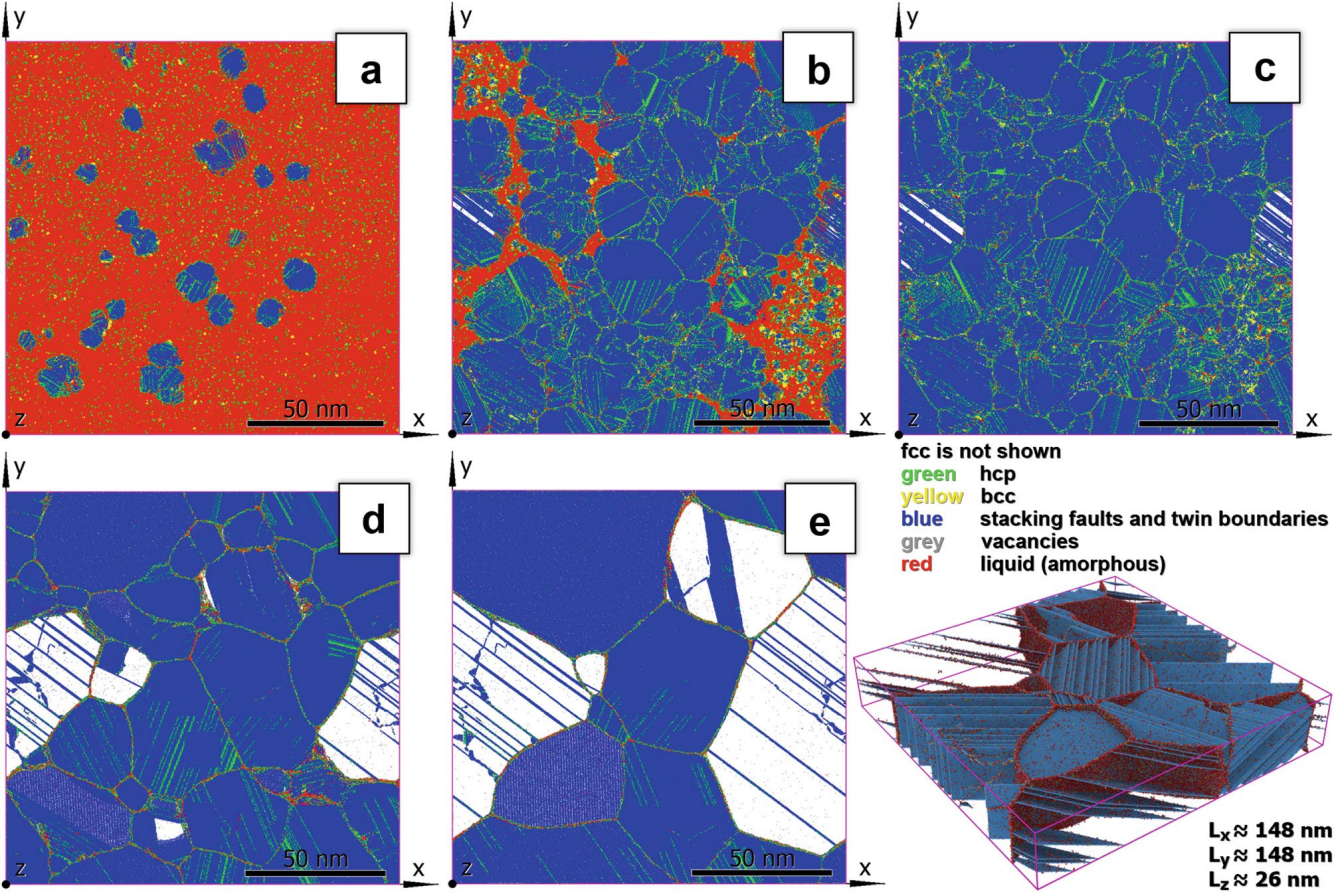
Nanocrystalline samples generated by crystallization from melt (M-samples). (**a**–**e**) Snapshots of the system structure at different stages: (**a**) The very beginning of fcc crystal nucleation in the liquid (the temperature of the sample at this instance is $T=900~K\approx 0.678T_m$), (**b**) Crystallization is almost completed—the liquid crystallized in a very fine-grained polycrystal (after this instance the temperature of the sample gradually increase to $T=1200~K\approx 0.904T_m$), (**c**–**e**) Illustration of grain coarsening during annealing at $T=0.904T_m$ (time interval 0–12 ns). No fcc structure atoms are shown. On the lower right, the isometric 3D view of the sample from (**e**) is shown.

The other option to construct nanocrystalline samples is to perform MD simulation of solidification from liquid (see Fig. 2). To achieve conditions for the formation of numerous crystallization embryos in the bulk of the MD sample, significant overcooling of liquid is needed due to the high surface free energy of crystal embryos in melt typical of metals^34^. On the other hand, there is a danger of getting a frozen amorphous glass-like structure from overcooled liquid in the time- and space-scales available for direct MD modeling which also depends on interatomic interaction potential being used in the simulations. In the present study for our samples of $\sim 45\times 10^6$ atoms and the EAM potential^30^ used, the onset of well-reproducible massive nucleation of fcc crystal embryos was achieved at $T\approx 0.68T_m$ at reasonable incubation times $\lesssim 1~ns$. The zero pressure melting temperature $T_m$ for the EAM potential^30^ was calculated to be $T_m=1328~K$ (the NIST experimental reference value is 1357.95 *K*) by two independent methods, namely, the Thermodynamic Integration Method^35,36^ in the formulation by Freitas et al.^37^ and modified Z-method^38,39^ which give essentially coinciding results.

**Figure 3:**
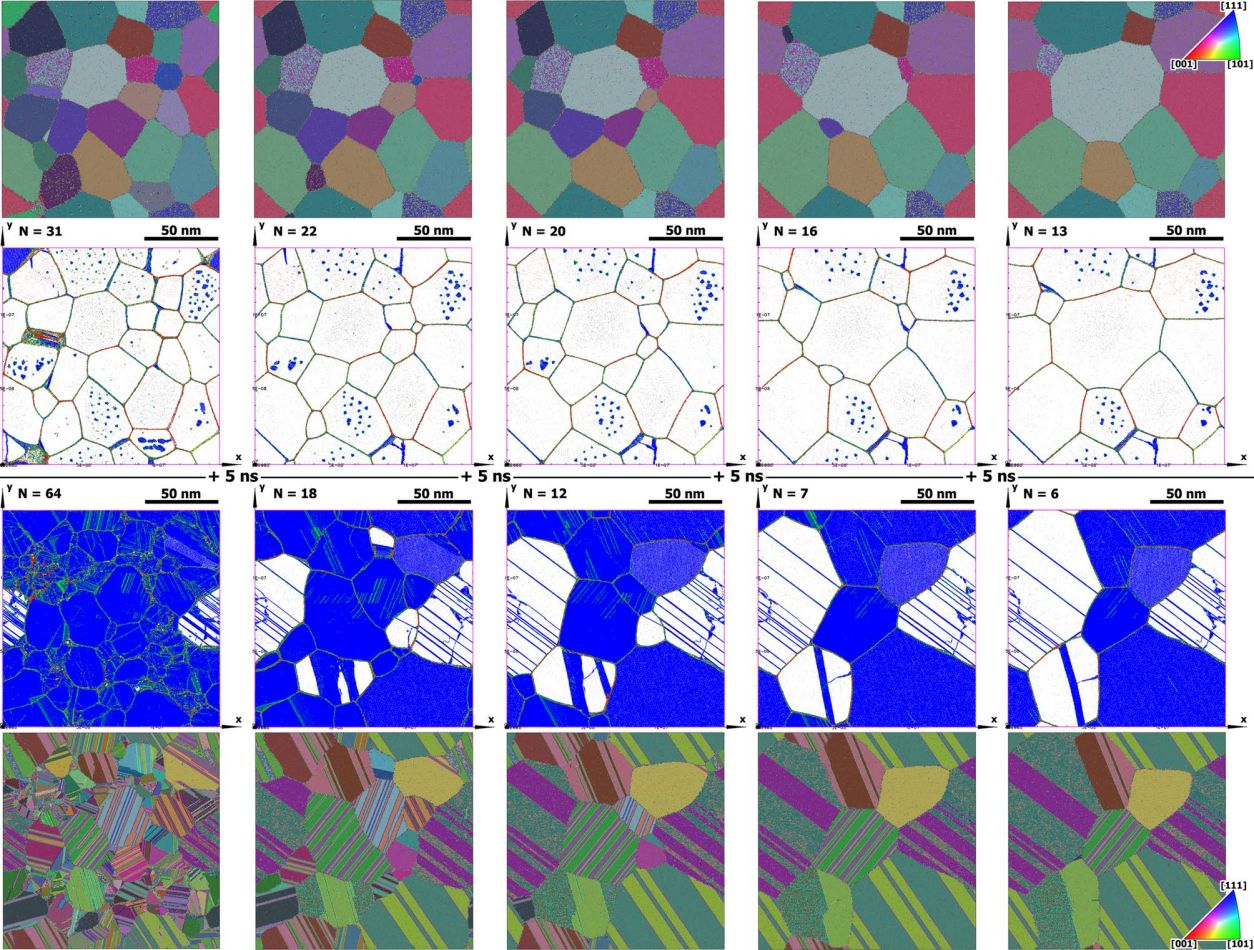
Comparison of the nanocrystalline grains and ingrain defect structures of V-type and M-type samples during annealing at $T=0.904T_m$. The first two rows are for the V-type sample while the last ones are for the M-type sample. The orientation colouring was done using PTM analysis in OVITO visualization software. The defect structure recognition was done by the ATA. The snapshots from left to right are separated by 5 *ns* time intervals. *N* is the number of grains in the samples at the presented instances.

Because of the random and non-simultaneous formation of crystallization nuclei in the overcooled liquid within the calculation box, it is difficult to control the number, shape, and relative size of resulting crystalline grains after the crystallization is completed. However, the following annealing at elevated temperature leads to recrystallization and grain coarsening (see Figs. 2 and 3). At this stage, for recrystallization acceleration the temperature is to be increased up to 1200 *K* which is $\sim 0.9~T_m$. The advantage of this technique is that grain shapes and boundaries between them are non-artificial unlike for the Voronoi cell filling method.

The samples crystallized from melt possess a high concentration of ingrain extended defects (numerous nanotwins, dislocations and stacking faults) due to the natural process of crystallization. Since atoms belonging to the fcc structure are not shown in Fig. 2 it seems (see Fig. 2a) that the stacking faults and twin boundaries (blue) originate immediately from liquid, but actually they are embedded in the growing fcc matrix. Because of the relatively low stacking fault energy in copper (experimental value is $\sim 40~mJ/m^2$^40,41^, which is well reproduced by the EAM potential^30^) the nanotwin boundaries and stacking faults dominate the other types of ingrain defects. In Fig. 2a–e they are seen from different angles—in some grains almost face up and from the edge in others (such grains look transparent). On the lower right of Fig. 2 the isometric 3D view of the sample from (e) is shown for clarity. During the annealing stage the number of defects significantly decreases but stays at a rather high level. The density of extended ingrain defects in such samples is in the range from $15\times 10^{-3}$ to $40\times 10^{-3}$, more than $90~\%$ of which are twin boundaries and less than $10~\%$ are stacking fault planes. Such samples imitate material after severe plastic deformation. Note here that Okita et al.^18,19^ used crystallization from melt to produce initial nanocrystalline samples. However, they studied iron which crystallizes in not close-packed bcc crystal structure. Because of that the authors unlike us got the initial ultrafine-grained samples essentially without extended ingrain defects. Apart from the extended ingrain defects during crystallization and following recrystallization there are vacancies and interstitial atoms in such samples. Concentrations of the vacancies and interstitials reach $10^{-4}$ and $10^{-5}$ respectively just after crystallization then decrease rapidly and stay nearly constant at the level $\sim 4.5\times 10^{-5}$ for vacancies and $\sim 2\times 10^{-6}$ for interstitial atoms during annealing at $T\approx 0.9~T_m$. The samples prepared with the crystallization-from-melt technique are designated hereafter as M-type samples. The samples of both types were constructed to have approximately the same size of $\sim 150\times 150\times 25~nm$ ($\sim 45\times 10^6$ atoms), the number of randomly orientated grains as well as random spatial distribution of grain centres, i.e. similar grain size distribution at the beginning of annealing (see Fig. 3).

## Results and discussion

**Figure 4:**
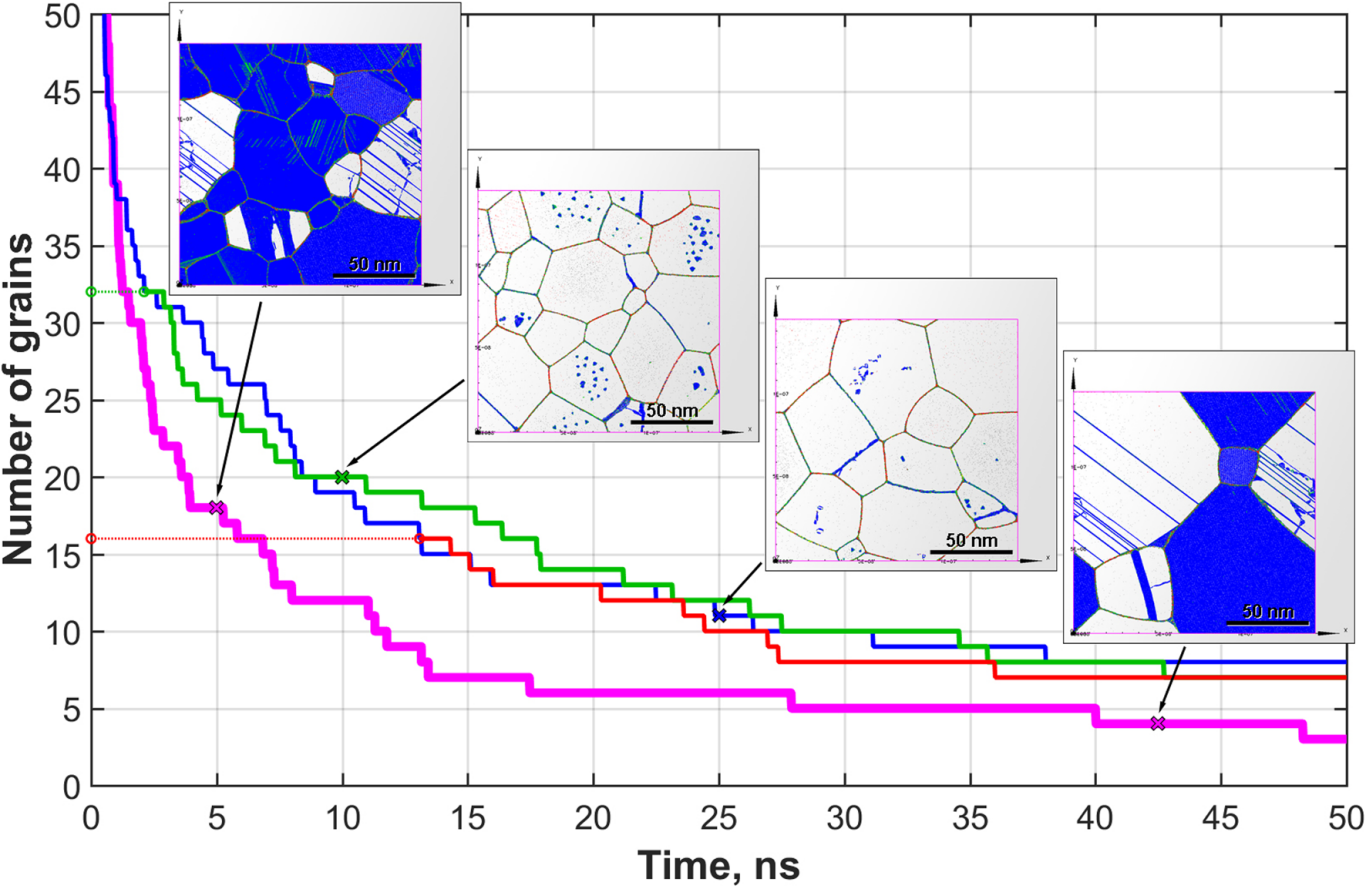
Grain coarsening upon annealing (number of grains in samples *vs.* time). The insets show the grain structures at different times. The thin solid lines are for samples of V-type with different initial numbers of grains: the blue, green, and red lines are for the V1, V2, and V3 samples (64, 32, and 16 initial grains, respectively). The last two lines are shifted to the times when the initial numbers of grains are equal to the current ones on the blue line for the V1 sample. The thick solid magenta line is for the sample of M-type.

Samples of both types (V and M) with an equal initial number of grains ($N=64$) were annealed at $T=0.904T_m$ for $\sim 50~ns$. Results of the simulations are presented in Fig. 4 as a dependence of the number of grains *N* on time. One can see that sample of V-type shows a lower rate of grain coarsening compared to that of the M-type sample. After 10 *ns* the number of grains is 20 and 12 in V-type and M-type samples, respectively, and after 25 *ns* of annealing the numbers are 11 and 6. Here we argue that the rate of grain growth for the samples of V-type depends on the current number of grains (i.e. the current average grain size) and does not depend on annealing history. One may also suppose that though the M-type and V-type samples are similar in average, the difference between their behaviour is due to peculiarities in their initial grain size and orientation distributions. To check the hypothesis, two more samples of V-type (V2 and V3) containing 32 and 16 initial grains were constructed. Their annealing curves are also presented in Fig. 4. The recrystallization rate curves for these samples were right shifted to the times 2.1 *ns* and 13 *ns* corresponding to current grain numbers in sample V1 equal to the initial number of grains in samples V2 and V3.

It is well seen in Fig. 4 that the annealing curves for all three V-type samples almost coincide, lie higher and demonstrate noticeably milder slope than the curve for the M-type sample. As for the sample of M-type, the rate of grain coarsening is determined not only by the current average grain size but also by the current concentration of ingrain defects. During annealing both the characteristics change. The question is why the M-type samples anneal faster.

Recrystallization is a kinetic process of metastable state decay and its rate is determined by a driving force which in its turn is defined by the degree of metastability and by the activation thresholds. Experimental results suggest that the higher thermal stability of ultradispersed structures is provided by the isotropic material structure^42^. Indeed, from the atomistic point of view, the presence, for example, of numerous low angle grain boundaries (i.e. preferred orientation) leads to lower thresholds of recrystallization. From this point of view both V- and M-type samples are isotropic because of the absence of the preferred grain orientation or texture at least at the beginning of modeling due to a rather big number of grains. On the other hand, the high concentrations of ingrain defects in M-samples increase their free energy and, therefore, the degree of metastability, thus providing for an additional driving force for recrystallization. So, the overall concentration of ingrain defects along with grain mutual orientations, sizes, and shapes is to be considered as an important factor affecting the thermal stability of submicrocrystalline materials. Though the behaviour is clear from the thermodynamic point of view, it would be interesting to look at the micro-mechanism responsible for the faster grain growth in the M-type sample.

According to the classical topological theory of recrystallization by von Neumann and Mullins^43,44^ the rate of the ideal grain growth is determined by the number of neighbouring grains (at equal other conditions such as grain boundary mobility and relative free energies). In quasi-2D case, a grain grows if the number of neighbouring grains is more than 6 (in 3D case, more than 16). It means that if at a triple boundary junction all three angles are equal to $2\pi /3$, such a configuration is quasi-steady. If one of the angles is greater, i.e. the ideal grain the angle belongs to has more than 6 neighbours, the junction point moves outward this grain due to the higher probability of atoms to “jump” into it, and as a result the grain grows. The motion of junction points results in the curvature of grain boundaries inward the growing grains (see Figs. 2 and 3) which in its turn increases the probability of atoms to jump (build-in) into this grain and, as a consequence, increases the growth rate.

**Figure 5:**
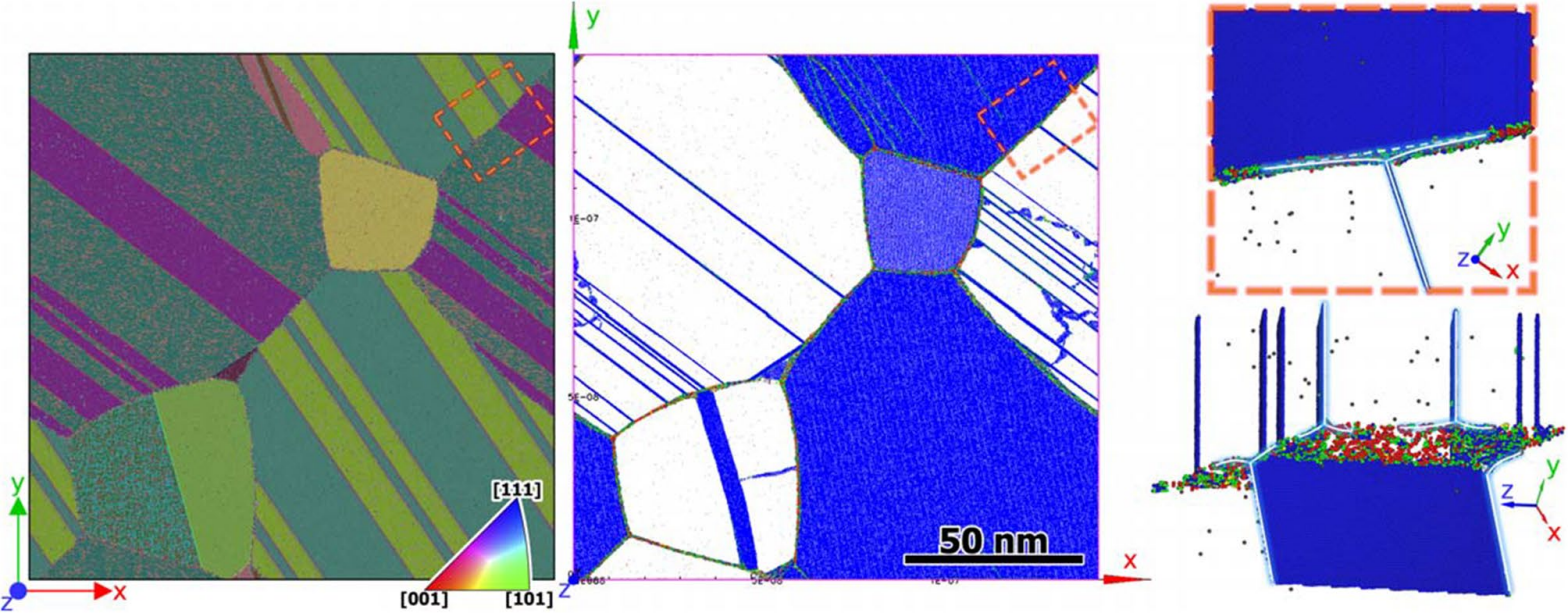
The grain orientation map from PTM analysis (on the left) and the defect structure recognized by the ATA (in the middle) in the M-type sample at $t=35~ns$. The enlarged defect structure in the box designated by the dashed rectangles is shown on the right. The blue color is for twin boundary atoms, the green is for atoms with hcp surroundings, the red shows atoms of unrecognized structure. The gray dots are vacancies in the fcc structure. Atoms belonging to the fcc structure are not shown. On the lower right, the same enlarged defect structure as on the upper right is shown tilted to demonstrate twin boundaries in the upper grain seen from the edge.

In Fig. 5 the PTM orientation colouring and the defect structure recognized by the ATA in the M-type sample at $t=35~ns$ are presented. An enlarged section of the grain boundary in the sample designated by the dashed rectangle is shown on the right in Fig. 5. One can see several twin boundaries resting against the grain boundary and causing its distortion. The grain boundary gets curvature and the junction points (actually junction lines because of the quasi-2D character of the sample) shift a bit along the twin boundaries. The atomic structure of the twin intersection with the grain boundary in nanocrystaline gold was reported in experimental paper^45^. In high-resolution transmission electron microscopy images (see Fig.3 in^45^) it can be seen that the twins indeed changed local curvature of the grain boundaries in the way described above. We argue that at equal other conditions the grains with the higher concentration of extended defects diminish faster due to this added curvature.

**Figure 6:**
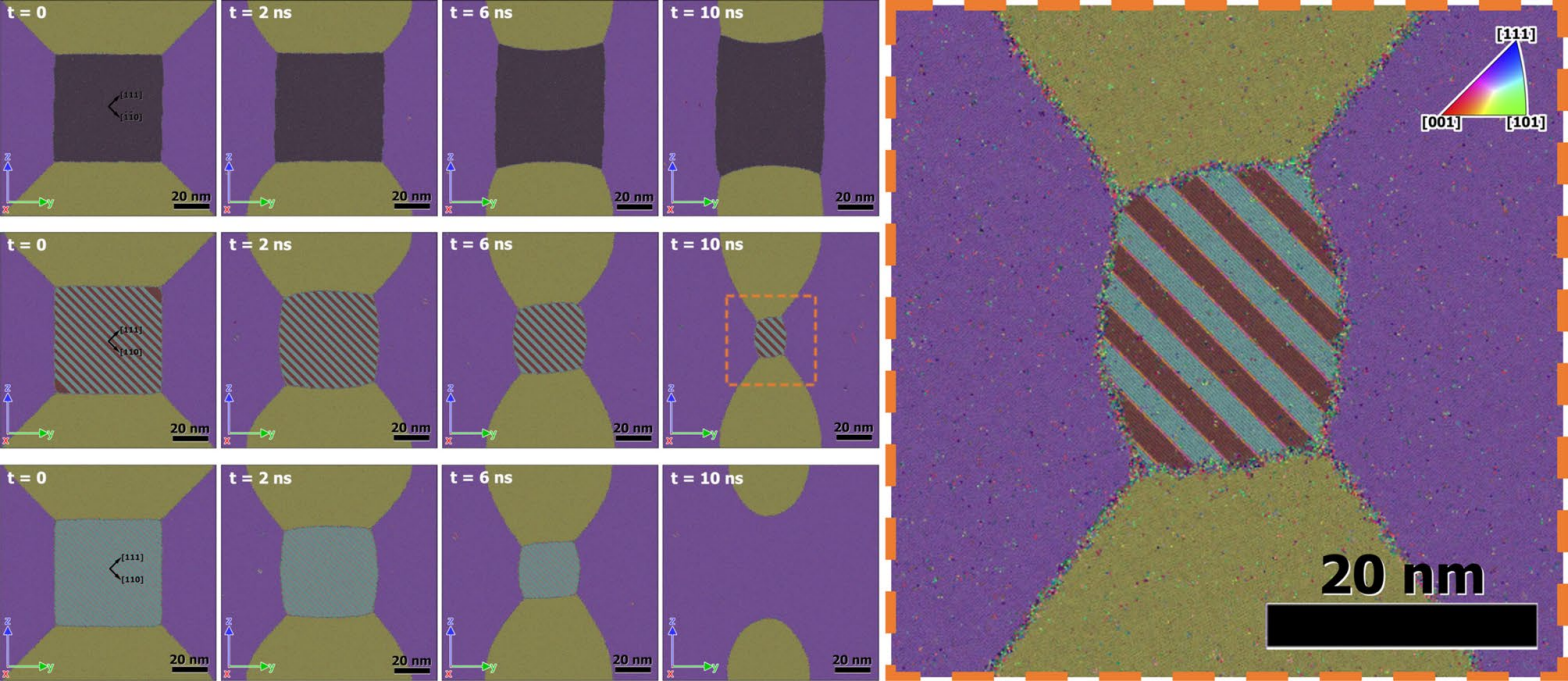
Recrystallization in artificially constructed systems of $\sim 17\times 10^6$ atoms in quasi-2D geometry ($\sim 14.4~\mathrm{nm}\times \sim 121~\mathrm{nm}\times \sim 121~\mathrm{nm}$) in periodic boundary conditions in all directions. On the left: snapshots of the system structures at $t=0$, 2, 6, and 10 *ns*. The first row is for the system free of ingrain defects. The second row is for the system with 38 twin boundaries (at $t=0$) in the central rectangular grain. The third row is for the system with 36 stacking fault planes (at $t=0$) in the central grain. Grain orientation colouring was done using PTM analysis. On the right, the enlarged central grain structure with nanotwins in the box designated by the dashed rectangle for $t=10~ns$ is shown.

**Figure 7:**
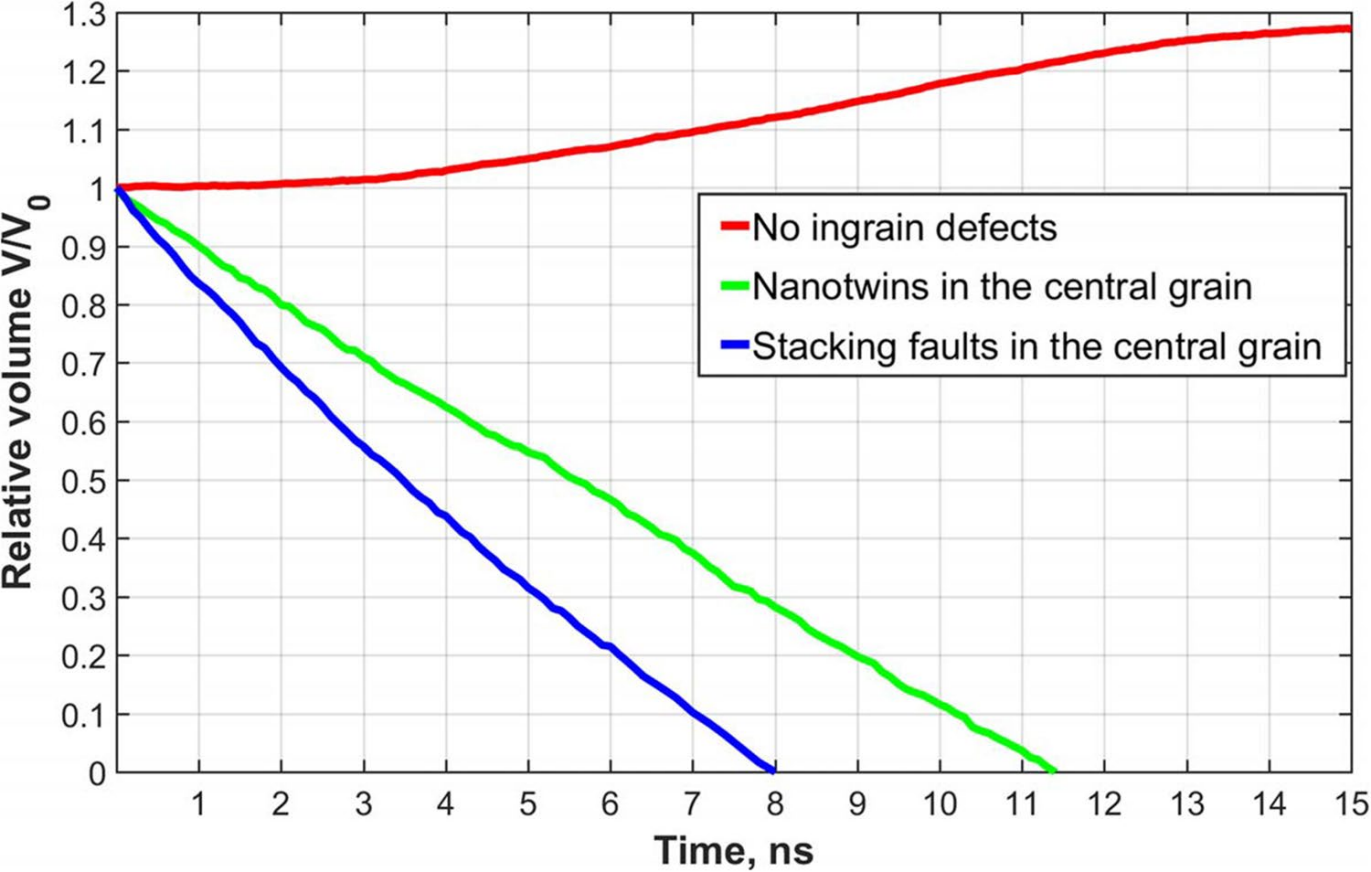
Relative volume $V/V_0$ of the central grain (see Fig. 6) *vs* time during the recrystallization in artificially constructed systems with the defect free central grain (red line), with the central grains containing nanotwins (green line) and stacking faults (blue line).

To check the hypotheses, some additional calculations were carried out using the LAMMPS MD code for artificially constructed grains in smaller systems of $\sim 17\times 10^6$ atoms in quasi-2D geometry ($\sim 14.4~\mathrm{nm}\times \sim 121~\mathrm{nm}\times \sim 121~\mathrm{nm}$) in periodic boundary conditions in all directions (see Fig. 6). A rectangular grain of initial size $\sim 60\times 60~\mathrm{nm}$ was put in the centre of the samples shown in the upper left corner of Fig 6. The first simulation was carried out with a system having no defects except the grain boundaries (the top row in Fig. 6). In the second simulation, 38 twin boundaries were added inside the central rectangular grain (the middle row in Fig. 6), and in the third simulation, 36 stacking fault planes were added in the central grain. Then the systems were annealed at $T=1200~K$ for 15 *ns* in *NPT*-ensembles. The relative central grain volume $V/V_0$ as a functions of time is presented in Fig. 7. One can see that the grains with nanotwins and stacking faults in the central grain show a rapid decrease of its size whereas the grain free of defects slowly grows. So, in this particular case, the presence of extended defects strongly affected the character and the rate of the central grain change. Note here also that stacking faults due to the higher free energy compared to that of twin boundaries have the greater effect. Nevertheless the samples with extended defects retain the anisotropy of central grain shrinkage proper to the defect free sample, i.e. slower rate of shrinkage in *z*-direction (see Fig. 6 right). But the anisotropy is weakened by the effects of twins. In Fig. 6 (right) it is also seen that twins changed geometry of the grain boundary forcing it to get curvature similar to that shown in Fig. 5 (right column). Twins subdivide the grain boundary into a number of convex segments which favor the grain boundary motion inward the grain. The general conclusion is that, indeed, the presence of extended ingrain defects favors the faster grain diminishing.

## Conclusion

State-of-the-art supercomputing technologies allow addressing the problem of materials design and their properties change due to processing, storage and exploitation in direct large-scale molecular dynamics simulations. In this paper the atomistic approach was applied to the simulation of thermal recrystallization of submicrocrystalline fcc material. It was proved that the timescale available for contemporary large-scale classical molecular dynamics ($\sim 10^2 ns$) was quite sufficient to track recrystallization at elevated temperatures in samples of representative sizes. Here the only factor affecting the thermal stability of nanocrystalline materials was considered, namely ingrain defects. The other factors such as alloying additions, impurities, and precipitates and their local distribution, texture *etc.* can also be studied with the technique. The effect of twins on grain growth in nanocrystalline gold under mechanical loading was experimentally studied in^45^. According to^45^ under the cyclic loading twins grow through grain boundary causing its local dissociation and thus assisting grain growth. This mechanism is quite different from that reported here for thermal grain growth that can be activated at rather high temperatures. As it was shown in the numerical experiments the ingrain defects weakened thermal stability and made the rate of recrystallization noticeably higher due to change of the grain boundary curvature making it locally convex and favorable for the inward motion. At the same initial number of grains and their average sizes, the dependence of the number of grains on time was steeper for the samples containing a lot of ingrain planar defects. Thus the conclusion following from the results of numerical experiments is as follows. To increase thermal stability, the technologies used for forming ultradispersed structures should be developed so as to avoid the thermomechanical processing regimes leading to the formation of structures with high concentrations of ingrain extended defects.

## References

[1] Meyers MA, Mishra A, Benson DJ (2006). Mechanical properties of nanocrystalline materials. Prog. Mat. Sci..

[2] Ackland G (2010). Controlling radiation damage. Science.

[3] van Swygenhoven H, Spaczer M, Caro A (1999). Microscopic description of plasticity in computer generated metallic nanophase samples: A comparison between Cu and Ni. Acta Mater..

[4] van Swygenhoven H, Farkas D, Caro A (2000). Grain-boundary structures in polycrystalline metals at the nanoscale. Phys. Rev. B.

[5] van Swygenhoven H, Derlet PM (2001). Grain-boundary sliding in nanocrystalline fcc metals. Phys. Rev. B.

[6] Hasnaoui A, van Swygenhoven H, Derlet PM (2002). Cooperative processes during plastic deformation in nanocrystalline fcc metals: A molecular dynamics simulation. Phys. Rev. B.

[7] Derlet PM, van Swygenhoven H (2002). Length scale effects in the simulation of deformation properties of nanocrystalline metals. Scrip. Mater..

[8] van Swygenhoven H, Derlet PM, Hasnaoui A (2002). Atomic mechanism for dislocation emission from nanosized grain boundaries. Phys. Rev. B.

[9] Hasnaoui A, van Swygenhoven H, Derlet PM (2002). On non-equilibrium grain boundaries and their effect on thermal and mechanical behaviour: A molecular dynamics computer simulation. Acta Mater..

[10] Bringa EM (2005). Ultra-high strength in nanocrystalline materials under shock loading. Science.

[11] Chen K, Wang L, Yin Y, Wang Q (2017). Molecular dynamics simulation of polycrystalline metal under high velocity nanoscale sliding. Chem. Eng. Trans..

[12] Chen D, Kulkarni Y (2018). Atomistic modeling of grain boundary motion as a random walk. Phys. Rev. Mat..

[13] Uehara T (2017). Molecular dynamics simulation of grain refinement in a polycrystalline material under severe compressive deformation. Mat. Sci. Appl..

[14] Liu D, Wang G, Yu J, Rong YK (2017). Molecular dynamics simulation on formation mechanism of grain boundary steps in micro-cutting of polycrystalline copper. Comp. Mat. Sci..

[15] Karavaev AV, Dremov VV, Ionov GV (2017). Atomistic simulations of dislocation dynamics in $$\delta $$-Pu-Ga alloys. J. Nucl. Mater..

[16] Karavaev AV, Dremov VV, Sapozhnikov F (2019). Shear strength of nanocrystalline $$\delta $$-phase Pu–Ga alloys: Atomistic simulations. J. Nucl. Mater..

[17] Dremov VV, Karavaev AV (2019). Atomistic simulation of strength properties of conventional and nano-structured materials. Proc. Manuf..

[18] Okita S, Shibuta Y (2016). Grain growth in large-scale molecular dynamics simulation: Linkage between atomic configuration and von Neumann-Mullins relation. ISIJ Int..

[19] Okita S (2018). Grain growth kinetics in submicrometer-scale molecular dynamics simulation. Acta Mater..

[20] Holm EA, Foiles SM (2010). How grain growth stops: A mechanism for grain-growth stagnation in pure materials. Science.

[21] Foiles SM (2012). Molecular dynamics simulation of grain growth in nanocrystalline Ni. Mater. Sci. Forum.

[22] Sapozhnikov FA, Dremov VV, Ionov GV, Derbenev IV, Chizhkova NE (2010). MOLOCH computer code for molecular-dynamics simulation of processes in condensed matter. EPJ Web Conf..

[23] Plimton S (1995). Fast parallel algorithms for short-range molecular dynamics. J. Comp. Phys..

[24] LAMMPS Molecular Dynamics Simulator. http://lammps.sandia.gov/.

[25] Larsen PM, Schmidt S, Schiotz J (2016). Robust structural identification via polyhedral template matching. Model. Simul. Mater. Sci. Eng..

[26] Stukowski A, Albe K (2010). Extracting dislocations and non-dislocation crystal defects from atomistic simulation data. Modell. Simul. Mater. Sci. Eng..

[27] Stukowski A (2014). Computational analysis methods in atomistic modeling of crystals. JOM.

[28] OVITO - Open Visualization Tool - Scientific data visualization and analysis software for atomistic simulation models in materials science and related disciplines. http://www.ovito.org/.

[29] Sapozhnikov FA, Ionov GV, Dremov VV (2008). An adaptive template method for analyzing crystal structures and defects in molecular dynamics simulations of high-rate deformations. Rus. J. Phys. Chem. B.

[30] Mishin Y, Mehl MJ, Papaconstantopoulos DA, Voter AF, Kress JD (2001). Structural stability and lattice defects in copper: Ab initio, tight-binding, and embedded-atom calculations. Phys. Rev. B.

[31] Nose S (1984). A unified formulation of the constant temperature molecular dynamics methods. J. Chem. Phys..

[32] Hoover WG (1985). Canonical dynamics: Equilibrium phase-space distributions. Phys. Rev. A.

[33] Uberuaga BP, Hoagland RG, Voter AF, Valone SM (2007). Direct transformation of vacancy voids to stacking fault tetrahedra. Phys. Rev. Lett..

[34] Sun Y (2018). Temperature dependence of the solid-liquid interface free energy of Ni and Al from molecular dynamics simulation of nucleation. J. Chem. Phys..

[35] Frenkel D, Ladd A. J. C (1984). New Monte Carlo method to compute the free energy of arbitrary solids. Application to the fcc and hcp phases of hard spheres. J. Chem. Phys..

[36] Frenkel, D. Free energy computation and first-order pase transitions. In *Molecular Dynamics Simulations of Statistical Mechanics Systems, XCVII*, 151–188 (Soc. Italiana di Fisica, Bologna, Italy, 1986).

[37] Freitas R, Asta M, de Koning M (2016). Nonequilibrium free-energy calculation of solids using LAMMPS. Comp. Mat. Sci..

[38] Wang S, Zhang G, Liu H, Song H (2013). Modified Z method to calculate melting curve by molecular dynamics. J. Chem. Phys..

[39] Karavaev AV, Dremov VV, Pravishkina TA (2016). Precise calculation of melting curves by molecular dynamics. Comp. Mat. Sci..

[40] Howie A, Swann PR (1961). Direct measurements of stacking-fault energies from observations of dislocation nodes. Philos. Mag..

[41] Cockayne DJH, Jenkins ML, Ray ILF (1971). The measurement of stacking-fault energies of pure face-centred cubic metals. Philos. Mag..

[42] Degtyarev MV, Chashchukhina TI, Voronova LM, Patselov AM, Pilyugin VP (2007). Influence of the relaxation processes on the structure formation in pure metals and alloys under high-pressure deformation. Acta Mater..

[43] von Neumann J (1952). Metal Interface.

[44] Mullins WW (1956). Two-dimensional motion of idealized grain boundaries. J. Appl. Phys..

[45] Luo X-M, Zhu X-F, Zhang G-P (2014). Nanotwin-assisted grain growth in nanocrystalline gold films under cyclic loading. Nat. Commun..

